# Conservation Education: The Signage Used in Eleven Swedish Zoos

**DOI:** 10.3390/ani16071113

**Published:** 2026-04-04

**Authors:** Elin Torgersson, Lina S. V. Roth, Maria Andersson

**Affiliations:** 1 Department of IFM Biology, Linköping University, SE-581 83 Linköping, Swedenlina.roth@liu.se (L.S.V.R.); 2 Department of Applied Animal Science and Welfare, Swedish University of Agricultural Sciences, P.O.Box 234, SE-532 23 Skara, Sweden

**Keywords:** zoo, signage, education, conservation, animal welfare

## Abstract

Education is one of the core roles of modern zoos, alongside research and conservation. In recent years, increasing emphasis has been placed on the importance of conservation within zoo education, and the term conservation education has become more widely used. Swedish zoos report using a variety of educational activities; however, systematic evaluations remain scarce. The aim of the present study was to analyse the content of species signage (*n* = 404) at 11 zoos in Sweden. A predetermined evaluation protocol was applied, including items related to conservation, animal biology, behaviour, ecology, and animal welfare, assessed using binary (“yes/no”) criteria. The signage analysis revealed that majority of signs provided basic species information, such as body size (88% of signs), lifespan (59%), geographical distribution (86%), diet (84%), and number of offspring (75%). In contrast, only 68% of signs included conservation status, 44% described specific threats, and just 17% provided information on actions visitors could take to support conservation. Information related to animal welfare was rare: 4% of signs mentioned enclosure design, 2% referred to enrichment, and only 1% included animal training. Overall, while Swedish zoos generally provided information on species ecology and, to some extent, conservation, guidance on how visitors could actively contribute to conservation was limited.

## Simple Summary

Education is one of the main roles of modern zoos, together with research and conservation. In recent years, zoos have placed more focus on teaching visitors about conservation, often referred to as conservation education. Swedish zoos use many different educational activities, but there are few studies that evaluate how effective these efforts are. This study aimed to examine the information presented on species signs at 11 zoos in Sweden. In total, 404 signs were analysed using a structured evaluation protocol. The protocol included topics such as conservation, animal biology, behaviour, ecology, and animal welfare. Each topic was recorded using simple yes or no criteria. The analysis showed that most signs included basic information about the species. For example, many signs described body size (88%), lifespan (59%), where the species lives geographically (86%), diet (84%), and number of offspring (75%). However, fewer signs included conservation information. Only 68% mentioned the conservation status of the species, 44% described specific threats, and just 17% told visitors what actions they could take to help conservation. Information about animal welfare was very limited. Only 4% of signs mentioned enclosure design, 2% referred to enrichment, and 1% described animal training. Overall, Swedish zoos provided general information about species and ecology, but fewer signs encouraged visitors to take action to support conservation.

## 1. Introduction

Education, together with conservation, research, and recreation, is widely regarded as one of the core roles of the modern zoo [1]. In many countries, zoos are also subject to legal requirements to provide education for their visitors, for example, the EU Directive [2]. In Sweden, zoos are required to educate visitors and raise awareness of conservation and biodiversity under the Swedish Species Protection Regulation [3]. In addition, the educational role of zoos is strongly emphasised by national and international zoo associations such as Swedish Association of zoos and aquariums (SAZA), European Association of zoos and aquariums (EAZA) and World Association of zoos and aquariums (WAZA) [4,5,6]. The EAZA Conservation Education Standards [7] state the aim “to mitigate the extinction of biodiversity through quality conservation education that raises awareness, connects people to nature, and encourages sustainable behaviours among the millions of people who engage with EAZA zoos and aquariums annually.”

Educational initiatives in zoos encompass a wide range of approaches, including signage, guided tours, keeper talks, and animal encounters. Signage is effective in encouraging behavioural changes, such as reduced noise levels [8]. Zager and Jensvold also found that signs can increase visitor engagement in relation to chimpanzees, suggesting that signage can contribute to achieving zoo education goals [9]. However, the content of zoo species signs has received limited attention in previous research. This study, therefore, focuses on analysing species signage in Swedish zoos, with particular emphasis on its relationship to conservation education.

A frequently cited argument in discussions of zoo education is that zoos worldwide receive approximately 700 million visitors annually, giving them a unique opportunity to educate the public about biodiversity and conservation issues [10]. This also places zoos in a position where they can contribute meaningfully to the 17 United Nations Sustainable Development Goals [11]. The SDGs form the core of the 2030 Agenda for Sustainable Development, adopted by all UN member states in 2015, and address social, economic, and environmental sustainability. Conservation education in zoos is particularly relevant to SDG 4, Quality Education, which aims to promote lifelong learning and ensure that learners acquire the knowledge and skills needed to support sustainable development. Given the large number of zoo visitors each year, zoos can serve as important platforms for education for sustainable development. Conservation education in zoos may also indirectly contribute to SDGs 15 (Life on Land), 14 (Life Below Water), and 13 (Climate Action). Furthermore, the WAZA Conservation Education Strategy emphasises that animal welfare should be prioritised whenever animals are involved in conservation education and highlights the importance of integrating animal care and welfare into educational initiatives [12].

Conservation education can take place in many different contexts, but what distinguishes zoos as educational settings is the presence of live animals. There is growing evidence that close, in-person experiences with live animals can positively influence zoo visitors’ interest in conservation [13] and their understanding of how they can contribute to the conservation of endangered species [14], and visitor–animal interactions [13].

Zoos function as informal learning environments, where visitors engage in free-choice learning that is often brief and self-directed. Research in educational psychology and conservation science suggests that increasing knowledge alone is rarely sufficient to promote pro-environmental behaviour; instead, behaviour is shaped by attitudes, social norms, and perceived behavioural control, as described in the “Theory of planned behavior” [15]. Indeed, while zoo visits can positively influence knowledge and attitudes, their impact on actual behaviour is often limited [16,17,18]. Consequently, conservation communication is more effective when it includes clear and feasible actions that enable individuals to contribute to conservation outcomes [16]. In this context, species signage can be understood not only as an informational tool but also as a potential means of influencing visitor behaviour.

This project aimed to evaluate the content of species signage used in Swedish zoos in relation to conservation education strategies. Species signs were therefore systematically analysed and, where possible, assessed in relation to conservation, animal biology, behaviour, ecology, and animal welfare.

## 2. Materials and Methods

All zoos located in Sweden that are members of the Swedish Association of Zoos and Aquaria (SAZA), with the exception of aquariums, were invited to participate in the study. In addition, one non-SAZA zoo was invited due to its status as one of the larger zoos in Sweden. In total, 13 zoos were invited, of which 11 accepted the invitation and participated throughout the entire project. Two zoos are situated in the northern part of Sweden, and two are situated in the southern part of Sweden. The remaining parks (7) are situated in the middle of Sweden.

### 2.1. Analysis of Zoo Signage

Data collection and analysis were conducted between September 2021 and January 2022. Personal communication with zoo staff indicates that signage is primarily updated in response to species turnover, rather than on a regular and systematic basis. All species signs, in Swedish or English, at the 11 participating Swedish zoos were analysed using a predetermined protocol. The protocol consisted primarily of binary (yes/no) items and recorded whether signage included information related to conservation, animal biology, behaviour (ethology), ecology, and animal welfare. The analysis was conducted at the species level, meaning that all signage related to a particular species and located at that species’ enclosure was treated as a single unit. If a sign contained at least minimal information on a given item, even if limited to a single sentence, it was recorded as “yes”. The protocol comprised 24 criteria organised into six thematic sections: (1) Threat status and conservation, (2) Biology, (3) Ethology, (4) Ecology, (5) Animal welfare in captivity, and (6) Other. The first section covered IUCN Red List status, threats to the species, conservation measures, the zoo’s own conservation contributions, and recommended visitor actions. The Biology section assessed whether basic life history information was provided, including body size, reproduction, and lifespan. Ethology addressed social behaviour, foraging, and stereotypic behaviours. The Ecology section covered geographic range, habitat, diet, ecological adaptations, and ecosystem role. Animal Welfare in captivity examined whether the signage described the enclosure, feeding, enrichment, and training practices at the zoo. Finally, the other section recorded whether information about specific individual animals was provided.

Qualitative data, such as certain text items in relation to conservation efforts suggested by zoos, were categorized into themes. Themes were identified during the analysis, and it was decided that each response could be categorized into more than one theme.

### 2.2. Data Analysis

Descriptive statistics were used to summarise the overall results of the study, using Microsoft Excel. Statistical analyses were performed using chi-squared tests in Minitab^®^, 21.3.1, Minitab, LLC, State College, PA, USA [19] to examine whether there were statistically significant differences between zoos. The chi-squared test compares observed frequencies with those expected by chance and was used to assess differences in qualitative characteristics of zoo signage across institutions.

## 3. Results

In total, across the 11 zoos that participated in the study, 404 species signs were analysed in relation to basic species facts, conservation, ecology, ethology, and animal welfare, over 313 unique different species. The five most common species were Lynx at 7 zoos (*Lynx lynx*), Wolverines at 6 zoos (*Gulo gulo*), Brown bear at 6 zoos (*Ursus arctos*), Wolf at 5 zoos (*Canis lupus*) and 7 zoos, and common seal (*Phoca vitulina*) at 4 zoos.

### 3.1. Basic Species Facts

**Figure 1:**
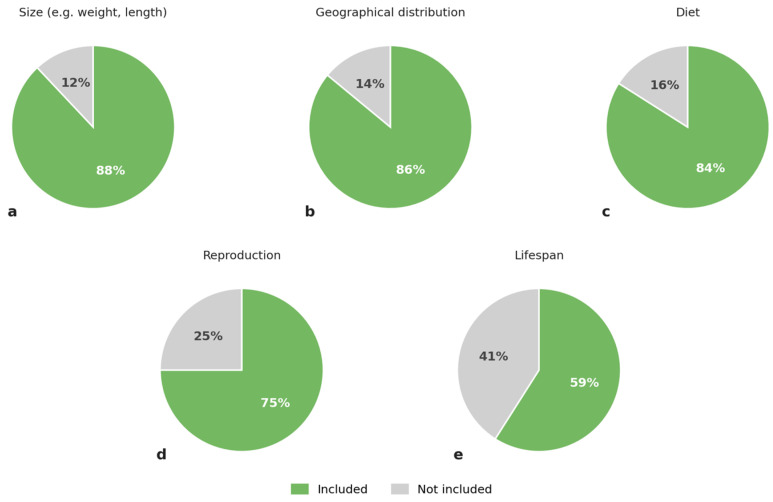
Percentage of zoo species signs include information about size (**a**), geographical distribution (**b**), diet (**c**), reproduction (**d**), and lifespan (**e**).

Approximately 88% (*n* = 354) of the 404 species signs included information about the size of the individuals belonging to a specific animal species, i.e., the expected weight, length, height or wingspan for individuals of the species (Figure 1a). Moreover, 86% (*n* = 349) of signage included information about the geographical distribution of the species (Figure 1b), and 84% (*n* = 341) included information about the diet of the species (Figure 1c). About 75% (*n* = 305) of species signs included information about reproduction (e.g., number of offspring or duration of gestation) (Figure 1d). And 59% (*n* = 237) of species signs included information about the lifespan of the species (Figure 1e).

### 3.2. Conservation

**Figure 2:**
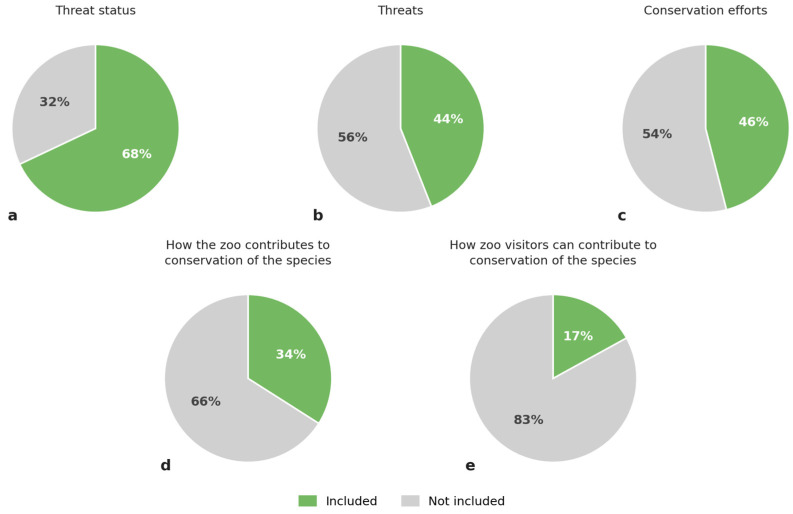
Percentage of zoo species signs including information about threat status (**a**), threats (**b**), conservation efforts (**c**), how the zoo contributes to conservation of the species (**d**) and how zoo visitors can contribute to conservation of the species (**e**).

**Figure 3:**
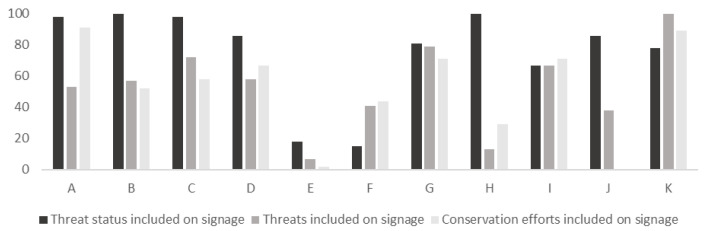
Percentage of species signs at each zoo (A–K), that included threat status, threats, and conservation efforts.

In total, 68% (*n* = 273) of the 404 species signs included information about the threat status of the species according to the IUCN Redlist [20], the Swedish Redlist or equivalent measure for domesticated species (Figure 2a). About 44% (*n* = 177) of the total number of species signs included information about the conservation threats (e.g., poaching, logging, agriculture, climate change) risking the survival of the species (Figure 2b), and 46% (*n* = 184) included information about conservation efforts aimed at the species (Figure 2c). There was a significant difference between individual zoos in relation to whether their signs included information or not about the following points: threat status (x^2^ = 233.2, *p* < 0.05) threats (x^2^ = 90.2, *p* < 0.05) and conservation efforts (x^2^ = 86.9, *p* < 0.05) (Figure 3).

A total of 24 different conservation efforts were identified on the signs, including ex- situ population management, reintroductions, research, education, monitoring of wild populations, protected areas, and the development of sustainable livelihoods for local people living within the species’ habitats (Table 1). Ex situ population management was the most frequently mentioned conservation effort, appearing on 137 signs across ten of the 11 zoos. This was followed by species protection, mentioned on 33 signs across seven zoos, and reintroduction projects, described on 19 signs across eight zoos.

About 34% of the 404 species signs (*n* = 136) included information about how the specific zoo contributed to conservation of the species (Figure 2d) and a total of eight different conservation efforts practiced by the zoos were identified, including ex situ population management, support of in situ projects, reintroduction, education and research (Table 2). Participation in ex situ population management programs was the most common conservation effort zoos mentioned on the signage to take part in, found on 135 signs at ten different zoos. This was followed by supporting in situ projects and participating in reintroduction programs, found on 12 and 10 signs, respectively, and both at six different zoos.

About 17% (*n* = 67) of the 404 species signs included information about how zoo visitors could contribute to conservation of the species (Figure 2e). This included a wide range of different actions ranging from donating money, to changing consumption or transportation habits, or report sightings of specific species (Table 3). A total of 21 different actions were identified. When measured by the number of signs, the most common action suggested was to become a species sponsor at the zoo, suggested on 44 signs; however, this action was only suggested at one particular zoo. Thus, given the number of zoos suggesting a certain action, donation was the most common action suggested, found on 11 signs in six different zoos. The results indicate a notable lack of suggested engagement activities in the signage, particularly for more active or behaviour-changing measures such as reporting sightings, reducing meat consumption, or participating in conservation programs (Table 3).

### 3.3. Ecology

Sixty-six percent (*n* = 268) of the 404 species signs included information about the species’ natural habitat. Twenty-three percent (*n* = 91) of the signs provided information about behavioural, anatomical, or physiological adaptations to the natural habitat (e.g., hibernation). Seven percent (*n* = 28) included information about the species’ ecological role and its importance within the ecosystem.

### 3.4. Animal Behaviour

In total, 59% (*n* = 239) of the 404 species signs included information about the social structure of the species (e.g., group-living, solitary). Social behaviours (e.g., communication between individuals of the species) were described in 20% (*n* = 81) of species signs, and foraging behaviours were described on 25% (*n* = 100) of species signs. Only 1 sign out of the 404 signs analysed included information about behavioural problems, such as stereotypical behaviours.

### 3.5. Animal Welfare and Animal Care in the Zoo

**Figure 4:**
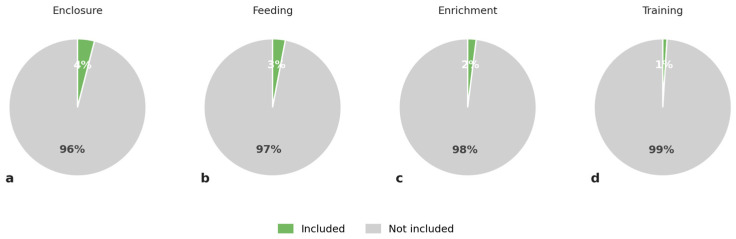
Percentage of zoo species signs including information about enclosure (**a**), feeding (**b**), enrichment (**c**) and training (**d**) in 11 Swedish zoos.

Four percent (*n* = 17) of the 404 species signs included information about the enclosure that that species was kept in (e.g., size of enclosure) (Figure 4a). Three percent (*n* = 12) of species signs included information about feeding of the species in the zoo (Figure 4b), and 2% (*n* = 9) included information about enrichment of the species in the zoo (Figure 4c). One percent (*n* = 5) of species signs included information about training of the species in the zoo (Figure 4d).

## 4. Discussion

The overall aim of this study was to assess the educational capacity of Swedish zoos in relation to conservation education strategies and animal welfare. While all zoos included in the study provided basic factual information about the species displayed, information on conservation was less consistently communicated, and information on animal welfare was largely absent. These findings indicate substantial scope for improvement.

Under the Swedish Species Protection Regulation, zoos are legally required to educate visitors and raise public awareness of biodiversity conservation, particularly by providing information about exhibited species and their natural habitats [3]. Signage represents one of the most widely used educational tools in zoos worldwide [21]. The present study demonstrates that Swedish zoos generally fulfil this obligation with respect to basic species information. For example, 88% of species signs included information on body size (e.g., weight, length, height, or wingspan), and 84% provided information on diet. In addition, 86% of signs described the geographic distribution of the species, and 66% included information about natural habitat. These results suggest that species signage plays a central role in meeting the legal requirements for zoo education in Sweden.

However, given the increasing emphasis on conservation within zoo education internationally, it is critical to examine not only whether conservation is addressed through signage, but also how it is communicated. The present study revealed significant differences among zoos in the extent to which their signage included information on threat status, specific threats, and conservation efforts. Across all zoos, 68% of signs included information on threat status, but fewer than half (44%) described specific threats facing the species. Similarly, only 46% of signs referred to conservation efforts, and a mere 17% provided information on how visitors could actively contribute to conservation. These findings align with previous research suggesting that conservation is often presented as the responsibility of zoos or external actors, rather than as an area in which visitors themselves can play an active role [22,23]. In line with these studies, the most frequently suggested conservation actions identified in the present study were donating money or becoming a species sponsor, approaches that risk distancing conservation from visitors’ everyday lives. To strengthen their conservation impact, zoos should critically review the conservation actions promoted through signage and other educational materials, prioritising actions that are both effective and realistically adoptable by visitors.

More than half of the signs included some information related to species ecology or behaviour. Nevertheless, information on adaptations, social organisation, or foraging behaviour appeared on only around 20% of signs, and behavioural problems or stereotypical behaviour were addressed on just a single sign. Information relating to animal welfare and animal care was particularly scarce. Only 3% of signs included information on feeding practices in the zoo, 2% referred to enrichment, and just 1% mentioned animal training. This lack of welfare-related content is notable, given that educating visitors about animal welfare and care has been highlighted as an important component of zoo education and conservation education in both empirical research and policy frameworks [12,24,25].

The reasons underlying the limited conservation information in signage likely reflect a combination of structural and institutional factors. Limited physical space on signage may constrain the amount and type of information that can be conveyed, potentially leading to a prioritisation of species identity and basic biological facts over more complex topics such as conservation measures or animal welfare in captivity. Additionally, the content of zoo signage may reflect deliberate institutional choices, where educational priorities are set at an organisational level rather than guided by evidence-based communication frameworks. A lack of formal training among those responsible for writing and designing signage may further contribute to the observed gaps, as sign writers may not be aware of which information categories are most effective in fostering visitor engagement and pro-conservation attitudes. Addressing these underlying factors, whether through institutional policy, staff training, or design guidelines, may therefore be as important as identifying what is currently missing from zoo signage.

The limited inclusion of animal welfare information suggests a missed opportunity for zoos to contextualise animal behaviour and management practices for visitors and to strengthen transparency and trust. Based on the results of the present study, we encourage Swedish zoos to prioritize improving their signage as a key educational tool, drawing inspiration from other zoos in visitor education and ex situ conservation programs. The present study provides a comprehensive analysis of the informational content of zoo signage, it does not capture visitor responses to or engagement with the signage. Future research should therefore incorporate visitor behaviour to assess how signage content influences awareness, attitudes, and conservation-oriented behaviour, as such insights would be critical for evaluating the true educational impact of zoo signage. It should also be noted, however, that this study focused exclusively on signage located at animal enclosures and does not capture the full range of educational communication within zoos, such as keeper talks, lectures, social media, or other interpretive materials.

## 5. Conclusions

Swedish zoos employ a wide range of educational media and activities. Based on an analysis of 404 species signs across 11 zoos, this study shows that signage is effective in communicating basic species information and likely contributes to fulfilling legal educational obligations. However, information related to species conservation, particularly actionable guidance for visitors, and animal welfare was limited. To enhance their educational impact, zoos should critically evaluate how conservation and animal welfare are communicated through signage and consider how these messages can better empower visitors to engage with conservation and understand animal care practices.

## Figures and Tables

**Table 1:** Conservation efforts mentioned on zoo species signage at 11 Swedish zoos (out of 404 signs).

Conservation Effort	Number of Signs	Number of Zoos
Ex situ population management	137	10
Species protection	33	7
Reintroduction	19	8
Research	14	8
Education	10	5
Monitoring of the wild population	8	5
Protected areas	7	6
Regulate hunting practices	5	4
Legislation	4	3
Financial compensation for livestock killed by predators/fishing gear damaged by seals	4	3
Stop poaching	3	3
Development of sustainable livelihoods	3	2
Collaborations between countries	3	1
Conflict mitigation	3	1
Rehabilitation	2	2
Wildlife corridors	2	2
Grants for the construction of predator-proof fencing	2	2
Politics	2	2
Hunting	2	2
Change logging practices	2	2
Restoring habitats	2	2
Fundraising	1	1
Stop illegal trade	1	1
Building a centre for sustainability	1	1

**Table 2:** Zoo conservation contributions mentioned on zoo species signage at 11 Swedish zoos (out of 404 signs).

How Does the Zoo Itself Contribute to Conservation	Number of Signs	Number of Zoos
Ex situ population management	135	10
Supporting in situ projects	12	6
Reintroduction	10	6
Collaborations with other organisations/companies	7	2
Education	6	4
Research	4	4
Selling products for the benefit of conservation	1	1
Fundraising	1	1

**Table 3:** Conservation actions suggested to zoo visitors on zoo species signage at 11 Swedish zoos (out of 404 signs).

Conservation Actions Suggested to Zoo Visitors	Number of Signs	Number of Zoos
Become a species sponsor at the zoo	44	1
Donation of money	11	6
Refrain from certain products	5	1
Suggestions for further reading	3	1
Report sightings of animals	2	2
Choose certified or eco-labelled products	2	2
Travel less by car	2	2
Use predator-proof fencing for domesticated animals	1	1
Talk about the issues of conservation	1	1
Vote in political elections	1	1
Support companies that reduce carbon dioxide emissions	1	1
Reduce meat consumption	1	1
Seasonal eating	1	1
Fly less	1	1
Support local transportation service	1	1
Participate in breeding programs	1	1
Do not leave litter in nature	1	1
Wash synthetic clothes less often	1	1
Dispose of waste properly	1	1
Report poaching	1	1

## Data Availability

The raw data supporting the conclusions of this article will be made available by the authors on request.

## Abbreviations

The following abbreviations are used in this manuscript:

SAZA	Swedish Association of zoos and aquariums.
EAZA	European Association of zoos and aquariums.
WAZA	World Association of zoos and aquariums.
SDG	Sustainable development goals.
